# Internet Addiction and Sleep Problems among Russian Adolescents: A Field School-Based Study

**DOI:** 10.3390/ijerph181910397

**Published:** 2021-10-02

**Authors:** Sergey Tereshchenko, Edward Kasparov, Marina Smolnikova, Margarita Shubina, Nina Gorbacheva, Olga Moskalenko

**Affiliations:** Research Institute of Medical Problems of the North, Federal Research Center “Krasnoyarsk Science Center of the Siberian Branch of the Russian Academy of Sciences”, 660022 Krasnoyarsk, Russia; kasparov_impn_1@mail.ru (E.K.); smarinv@yandex.ru (M.S.); marg-shubina@mail.ru (M.S.); n-n-gorbacheva@yandex.ru (N.G.); olga_olgaol@mail.ru (O.M.)

**Keywords:** adolescents, Russia, Internet addiction, game addiction, social media addiction, sleep problems, daytime sleepiness

## Abstract

This study aims to establish a link between disturbances in the night sleep habitus, quality of sleep, and daytime sleepiness in adolescents with Internet addiction and different types of content consumed. Methods: This is a cross-sectional observational study of a school sample in three large cities in Central Siberia. 4615 schoolchildren of 12–18 years old were examined. The Russian-language versions of the Chen Internet Addiction Scale, the Game Addiction Scale for Adolescents, and the Social Media Disorder Scale were used to identify Internet addiction. Questions from the Pittsburgh Sleep Quality Index questionnaire were used to assess nighttime sleep. Daytime sleepiness was assessed using the Pediatric Daytime Sleepiness Scale questionnaire. Results: Adolescents with Internet addiction go to bed and wake up late; they are characterized by a decrease in the duration of nighttime sleep, an increase in sleep onset latency, and frequent nighttime awakenings, as well as more pronounced daytime sleepiness. Among the sleep parameters studied, the indicators of daytime sleepiness and night awakening scales have the highest effect size in Internet-addicted adolescents, regardless of the media consumed. Conclusion: Internet-addicted adolescents are characterized by significant disturbances in the quality of nighttime sleep and excessive daytime sleepiness, which requires appropriate psychological correction.

## 1. Introduction

The last two decades have been characterized by an avalanche-like increase in the prevalence of Internet use in all social groups, especially among adolescents and young adults [1]. In Russia, 80.9% of the population use the Internet, which is 16% of European users—the highest value among all European countries [2]. Moreover, over the past 10 years, the number of users has almost doubled—in 2010, the penetration of the Internet into the Russian population was only 42.8% [2]. A certain number of Internet users, mainly adolescents and young adults, develop Internet addiction (IA) or “pathological/compulsive use of the Internet”, characterized by a loss of control over the time spent online, an obsessive craving for various types of Internet activities, which often becomes the catalyst for the formation of a wide range of psychosocial and psychosomatic problems.

Even though the common definition and diagnostic criteria of IA are continuously under debate, psychologists and psychiatrists have agreed on the four components essential to this diagnosis [3,4,5].

(1) Excessive Internet use (especially when characterized by loss of time or neglecting basic functions): compulsive striving for Internet usage, growing importance of Internet in an adolescent’s system of personal values;
(2) Withdrawal symptoms: mood swings (abstinence withdrawal symptom) when Internet is unavailable (anger, depression, and anxiety);
(3) Tolerance: need to spend increasing amounts of time on the Internet, exemplified by the need for increased use of the Internet to relieve negative emotional symptoms; and
(4) Negative consequences: excessive engagement in Internet use, contrary to negative psychosocial outcomes; loss of previous hobbies and entertainments as a result of such engagement; loss of social relations and educational, and sport opportunities resulted from undue usage of Internet; quarrels and lies with regards to using the Internet; relapse: self-control failure in relation to Internet use.

Based on the above general diagnostic criteria, in recent years, questionnaires have been developed and validated for various adolescent populations to assess general IA (undifferentiated by consumed content) [6], as well as special questionnaires to assess gambling addiction [7] and dependence on social networks [8].

There is a reasonable opinion that the formation of a full deployed IA complex, or pathological Internet use, is preceded by maladaptive Internet use (MIU); when a teenager already has some IA signs, but not all of them, such teenager is an at-risk user [9,10,11,12]. However, psychological and psychosomatic problems may arise at this stage, which has been shown by some works, including our previous studies [10,13,14,15].

In recent years, it has become apparent that excessive human interaction with information and communication technologies is becoming a major public health concern. In particular, it has been shown that IA in adolescents is associated with serious psychological disorders and social difficulties. Numerous studies convincingly demonstrated IA comorbidity with a broad range of psychopathologic conditions. Ho et al. in their meta-analysis demonstrate IA comorbidity with depression (OR = 2.77, CI = 2.04–3.75), anxiety disorders (OR = 2.70, CI = 1.46–4.97), and attention deficit–hyperactivity disorder (ADHD); (OR = 2.85, CI = 2.15–3.77) [16]. In their systematic review, Carli et al. showed that depressive disorders and ADHD have the strongest association with IA. A lesser but still meaningful association was found with anxiety, obsessive compulsive disorders, social phobia, and aggressive behavior [17]. The same conclusions were supported by another systematic review [18]. Durkee’s et al. [19] research involved a representative sample of 11,356 adolescents from 11 European countries and found that IA is associated with self-destructive and suicidal behavior as well as depression and anxiety. The same results were obtained by Jiang et al. [20]. Other investigators proposed that IA is associated with definite personal features, namely “sensation seeking”. This is frequently described by Western authors as a striving for new, unordinary, and complicated sensations, which are often risky [21]. In their longitudinal study, Guillot et al. demonstrated IA associations with anhedonia in adults (i.e., weakened ability to feel pleasure, which is typical for depressive disorders) [22].

IA associations with psychosomatic diseases are not clear, though they might be possible given that comorbid factors may be mutually connected (e.g., anxiety, depressive, and obsessive- compulsive disorders). Wei et al. found that IA is associated with chronic pain syndromes [23]. Cerutti et al. found no statistically meaningful associations between IA and tension headaches/migraines, although somatic pain symptoms, in general, were frequently found in IA patients [24].

It was found that IA in adolescents is associated with chronic conditions (OR = 1.58 CI = 1.11–2.23), back pain (OR = 1.46 CI = 1.04–2.05), overweight (OR = 1.74 CI = 1.03–2.93), musculoskeletal pain (OR = 1.36 CI = 1.00:1.84), and sleep disorders (OR = 2.16 CI = 1.62–2.88) [25]. However, when analyzing the general model, only the association with sleep disorders remained statistically significant [25].

There is growing evidence of a link between IA and various types of sleep disorders. In modern industrial society, filled with information flows and gadgets, IA is becoming one of the leading causes of the development and increase in the severity of sleep disorders [26], including among adolescents [27]. A large number of studies show a pronounced mutual influence of IA and sleep disorders. Thus, excessive time spent online reduces the required night sleep of schoolchildren [28], is associated with going to bed later [29], various nighttime sleep problems [26,30], including insomnia [31], as well as weakness [32] and sleepiness during the day [33]. It has been shown that exposure to light from monitors, especially the blue spectrum, is related to the suppression of melatonin secretion and, probably, associated difficulties in falling asleep, which may exacerbate the pathological effect of excessive online time on nighttime sleep [34,35].

Chen and Gau in their longitudinal study of schoolchildren based on the parental Sleep Habit Questionnaire showed an association of IA with insomnia, especially at the beginning and middle of nighttime sleep, with subsequent disturbance of the circadian rhythm [36]. It seems interesting that the authors established bi-directionality in the interaction of IA and night sleep disorders: initially, dyssomnia is a risk factor for addiction, and later, the already developed IA leads to a disruption of the circadian rhythm. Kawabe et al. recorded that going to sleep later and awakening later, a decrease in total sleep duration, and an increased general index of sleep problems according to the Child and Adolescent Sleep Checklist in adolescents with IA [27]. A large number of studies show a significant decrease in the quality of nighttime sleep, verified using the Pittsburgh Sleep Quality Index (PSQI) questionnaire [37,38,39,40,41]. Besides, daytime sleepiness was increased in adolescents with IA [37,38,39,40].

There is a systematic review and pooled meta-analysis of studies on the relationship between IA and sleep disorders. In 2014, Lam, in his systematic review, demonstrated a significant amount of convincing evidence for the association of IA with sleep impairment and insomnia [30]. A recent (2019) meta-analysis of 23 studies in this direction, carried out by Alimoradi et al., quantitatively assessed the strength of such associations and showed a significant risk of dyssomnia in the case of IA (OR = 2.20; 1.77–2.74), as well as a significant reduction in night sleep [26].

The general prevalence of IA and its structure depending on the media consumed (dependence on video games/social networks), as well as the psychosomatic consequences of such dependence in Siberian adolescents, was previously presented by the authors in a series of publications [13,14,15]. It was shown that the prevalence of IA, verified by the Chen Internet Addiction Scale (CIAS) questionnaire, in adolescents from Krasnoyarsk was 6.8%, game addiction was more common in males, and addiction to social networks in females. An association of IA with adolescent psychosocial problems was established and then verified using the Strengths and Difficulties Questionnaire. A pronounced comorbidity of IA with recurrent cephalalgia, dorsalgia, and recurrent abdominal pain was demonstrated.

This study aims to establish a link between disturbances in the nightly sleep schedule, quality of sleep, and daytime sleepiness in adolescents with Internet addiction and different types of media consumed. We hypothesize that Internet-addicted adolescents in Central Siberia, as well as in other previously surveyed populations [26,30], will have sleep disorders, in particular, with respect to the total duration of nighttime sleep and the severity of daytime sleepiness. In addition, we expect to find the characteristics of sleep disorders depending on the content consumed by adolescents, age, and gender differences.

## 2. Materials and Methods

### 2.1. Participants

This is a single-point cross-sectional observational study, a kind of case-control school sample in three large cities in Siberia. The research subjects were adolescents aged 12–18 years old (46.2% were male (average age—14.57 ± 1.55 years old) and 53.8% were female (average age—14.58 ± 1.55 years old); average age of total sample—14.58 ± 1.55 years old)—students of 10 general education schools in Krasnoyarsk (*n* = 3084), 4 general education institutions in Abakan (*n* = 1314), and 2 general education schools in Kyzyl (*n* = 217).

### 2.2. Measurement

After receiving an informed parental consent, students were notified of voluntariness and confidentiality of the study and asked to complete paper versions of self-report questionnaires within 45 min in a common classroom. The survey was conducted in the spring of 2019. The study was approved by the Ethics Committee of the Federal Research Center “Krasnoyarsk Science Center of the Siberian Branch of the Russian Academy of Sciences”.

#### 2.2.1. Internet Addiction Measurement

The internationally accepted CIAS scale [6], adapted by Malygin and Feklisov [42] was used to study the presence of Internet-addictive behavior. The CIAS covers five symptomatic criteria for addictive behavior, which include compulsive symptoms, withdrawal symptoms, signs of tolerance, psychological or physical problems, and difficulty in time management. The questionnaire includes 26 statements, each of which is assessed on the 4-Point Likert’s Scale: “Not Suitable At All” (1 point), “Poorly Suitable” (2 points), “Partially Suitable” (3 points), and “Completely Suitable” (4 points). An overall CIAS score of 27 to 42 was assessed as adaptive Internet use; 43–64 points—maladaptive Internet use; 65 points and above—IA.

The analysis of the structure of content consumed by adolescents with IA was carried out using the Russian-language version of the questionnaire for assessing game addiction, “Game Addiction Scale for Adolescents” [7] and the questionnaire of social network addiction, “The Social Media Disorder Scale” [8]. The questionnaire for assessing game addiction consists of 7 questions concerning behavioral disorders in adolescents caused by excessive interest in Internet games. Each of the questions is rated on the 5-Point Likert Scale: “never” (0 points), “rarely” (1 point), “sometimes” (2 points), “often” (3 points), “very often” (4 points). A diagnosis of game addiction was made when the total score of 3–5 scales of the questionnaire was 12 points or more. The Social Media Addiction Questionnaire consists of 9 questions regarding behavioral disorders caused by the overuse of social media. Each question has two possible answers: “no” and “yes”. For each “Yes” answer, 1 point is assigned. An overall score of 5 or more points indicates an addiction to social media.

#### 2.2.2. Sleep Parameters Measurement

To assess the quantitative and qualitative characteristics of night sleep, the authors used questions from the Pittsburgh sleep quality index (PSQI) questionnaire [43]. The adolescents were asked the following questions:

1. What time did you usually go to bed during the last month on school days (excluding weekends)?
2. ow much time (how many minutes) did it usually take you to fall asleep (during the last month)?
3. What time did you usually wake up during the last month on school days (excluding weekends)?
4. During the past month, how often have you had trouble sleeping because you woke up in the middle of the night or in the morning?

The last question was estimated in points, the points were summed up to quantify the frequency of night awakenings:

- Not once within the last month—0 points
- Less than once a week—1 point
- Once or twice a week—2 points
- Three or more times a week—3 points.

To assess the degree of daytime sleepiness, the Pediatric Daytime Sleepiness Scale (PDSS) questionnaire developed by Drake et al. [44] was used. The questionnaire consists of 8 questions concerning daytime sleepiness. Each of the questions is evaluated on the 5-Point Likert Scale: “never” (0 points), “rarely” (1 point), “sometimes” (2 points), “often” (3 points), “very often” (4 points). The points of all questions, except the third one, are summed up with the direct score; the third question is scored in reverse. Higher values of the total score correspond to greater severity of daytime sleepiness.

### 2.3. Statistical Analysis

The statistical analysis of the results obtained was carried out with the use of Statistica v.12.5 (Stat Soft Inc., Tulsa, OK, USA). The minimum required sample size (*n* = 132) was calculated, taking into account the likely prevalence of IA at 10%, published data on possible differences (the total duration of night sleep was 5.38 ± 1.89 and 7.19 ± 1.21 h in adolescents with IA and without it, respectively, in the study by Kawabe et al. [27]) at a statistical power level of 95% and α = 0.001. The distribution type was determined using the Shapiro-Wilk test. Student’s t-test was used in the case of normal distribution in the comparison groups. In the absence of signs of normal distribution, the nonparametric Kruskal–Wallis H (for three comparison groups) and Mann–Whitney U (for pairwise comparison) tests were used to assess the differences in the groups. Effect size (Cohen’s d) for Kruskal–Wallis H and Mann–Whitney U tests were calculated according to recommendations by Fritz, Morris & Richler [45] with the public domain software https://www.psychometrica.de/effect_size.html (accessed on 2 October 2021). The strength of the relationship between the two variables was determined using the Spearman’s rank correlation coefficient (r). The confirmatory factor analysis was carried out in the statistical computing system R (R Foundation for Statistical Computing, Vienna, Austria) [46], the lavaan package [47], implemented in the Jamovi graphical interface (ver. 2.0 [48]). The quality of the models’ fit with empirical data was assessed using the following indicators: χ2, root mean square error of approximation (RMSEA), comparative fit index (CFI), Tucker–Lewis index (TLI). The comparison of groups by the qualitative binary feature was performed using the Pearson χ2 test. The data are presented as “Arithmetic mean ± Standard Deviation (SD)” in the case of using parametric criteria and the Median (25 quartile—75 quartile)—in the case of using nonparametric criteria.

## 3. Results

The demographic characteristics for the sample of adolescents in this study and the main statistics of the IA parameters and sleep scores used in the study are presented in Table 1. The psychometric characteristics of the questionnaires used in our general sample are shown in Table 2.

### 3.1. The Prevalence of Internet, Gaming and Social Media Addiction

In the general sample, the prevalence of total IA, assessed by the CIAS questionnaire, was higher in girls than in boys (*p* < 0.001), which somewhat contradicts the data of most other studies [49]. Perhaps this is due to gender characteristics of the content consumed by Siberian adolescents: girls were three times more likely to be addicted to social networks, while boys were two times more likely to be addicted to gambling (Table 1). In addition, girls got up earlier, spent less time sleeping at night and had increased sleep onset latency, frequency of night awakenings and daytime sleepiness (Table 1).

### 3.2. Relationship between Internet Addiction and Sleep Problems According to Age and Gender

Since both the features of sleep schedule and the IA phenomenon have characteristic distinctive features depending on the age and gender of respondents in our and other samples [14,49], we carried out a separate analysis of sleep parameters in the groups of younger (12–14 years old) and older (15–18 years old) adolescents with additional gender stratification.

The results of the analysis of the relationship between the parameters of nighttime sleep and daytime sleepiness with the general IA (undifferentiated by consumed content), verified by the CIAS questionnaire, are presented in Table 3 and Table 4. The data obtained indicate that adolescents with IA have pronounced features of qualitative and quantitative sleep characteristics for all the parameters selected for the analysis and in all age–gender groups.

Internet-addicted adolescents went to bed later and woke up later, but their total duration of nighttime sleep was significantly lower than that of adolescents with adaptive Internet use.

At the same time, it was more difficult for adolescents with IA to fall asleep—sleep onset latency was statistically significantly higher in IA groups, independently of age and gender. Moreover, they clearly showed symptoms of late insomnia—they woke up more often in the middle of the night and in the morning, which was recorded using the higher values of the night awakenings. These features of nighttime sleep in adolescents with IA were obviously accompanied by higher values of the daytime sleepiness scale calculated using the PDSS questionnaire.

Among sleep characteristics, the highest effect size exhibited the PDSS score with Cohen’s d 0.7–0.8 in all age–gender groups. The second significant parameter was weekdays bedtime with Cohen’s d 0.4–0.7, although an increase in the time of going to bed was accompanied by reductions in the total duration of night sleep with moderate Cohen’s d (0.3–0.4) only in older adolescents.

### 3.3. Relationship between Gaming/Social Media Addiction and Sleep Problems by Age and Gender

Data on the relationship between sleep parameters and verified addictions to Internet computer games and social networks are presented in Table 5, Table 6, Table 7 and Table 8, respectively.

As in the case of using the CIAS questionnaire, the addiction had the greatest effect on the severity of daytime sleepiness, both for Internet game addiction and social media addiction. The presence of Internet game addiction had the biggest effect size on sleep parameters for boys, especially younger ones, with low-to-moderate Cohen’s d (0.2–0.6). For example, the higher frequency of night awakenings was typical only for boys, but not for girls with Internet game addiction. Among Internet game overusers sleep onset latency was higher only in younger (12–14 years old) adolescents, both in boys and girls. Social media addiction had practically no effect on the quantitative parameters of nighttime sleep in all age-gender groups and had a very weak effect on the qualitative characteristics of nighttime sleep. Among social media overusers the frequency of night awakenings was higher in almost all age–gender groups with low Cohen’s d (0.2); sleep onset latency was slightly higher only in girls but not in boys.

### 3.4. Correlation Analysis

The correlation analysis of the scales used in the study questionnaires and quantitative sleep parameters is presented in Table 9. A moderate degree of correlation of the CIAS scale with the GASA and SMDS scales (Spearman’s r = 0.415 and 0.608, respectively) was revealed. Among the sleep parameters, the highest association with all the used IA scales is demonstrated by the severity of daytime sleepiness, assessed by the PDSS questionnaire. Moreover, the highest strength of the relationship was found in the CIAS questionnaire (r = 0.422), an intermediate position was occupied by the social network dependence questionnaire (r = 0.357), and the lowest strength of the relationship was recorded for the game addiction questionnaire (r = 0.198). High values of the daytime sleepiness scale were also positively associated with sleep onset latency (r = 0.194) and negatively—with the total duration of nighttime sleep (r = –0.311).

## 4. Discussion

It is well-known that at secondary school age, for proper rest, the duration of nighttime sleep should be at least 8 h [50,51]. Schoolchildren of 12–14 years old in the sample generally fulfilled this recommendation; among older adolescents in all age–gender groups, the duration of nighttime sleep was less than the recommended 8 h, with the lowest values in groups with IA—6.4 h among males and 6.6 h among females (Table 3 and Table 4). It should be mentioned that even in the lack of addiction in this age group, sleep duration did not exceed 8 h. Lack of sleep has been seen in other adolescent populations as well [52]. For example, Norwegian schoolchildren aged 16–17 sleep on average 7 h 36 min on school days [53]; sleep duration of fewer than 8 h at this age was also recorded in Poland, Latvia, Estonia, and Greece [52]. However, in the overwhelming majority of European countries and the USA, older students sleep the recommended 8 h or more [52]. The revealed insufficient amount of nighttime sleep in older adolescents from Central Siberia, especially in the case of IA, should attract the attention of specialists, since the deficit of nighttime sleep is associated with several mental and somatic problems, as well as with a decrease in school performance [54,55].

It is noteworthy that non-adaptive Internet users are characterized by difficulties in falling asleep—in all selected groups, sleep onset latency (the actual equivalent of early insomnia) progressively increased with an increase in the degree of IA. It was previously found that an increase in this time in adolescents was characteristic when they had felt a large number of exciting emotions during the day—the repeated mental experience of which did not allow adolescents to fall asleep quickly [56]. Moreover, it has been shown that an increase in sleep onset latency is an indirect reflection of many psychosocial problems: depression [57], emotional, and behavioral problems [58], decreased academic performance [59], weakness, and sleepiness during the day [57,60].

Nighttime awakenings, as an equivalent of late insomnia, were also significantly more frequent in the groups of adolescents with non-adaptive Internet use and IA, with a slightly higher frequency in females. The data of this study are consistent with the results of other studies in this direction. For example, in the study by Canan et al. in 16-year-old Internet-addicted adolescents, along with a decrease in the time of nighttime sleep, frequent night awakenings were also recorded [61]. A recent study of Turkish adolescents also showed a decrease in the duration of nighttime sleep, an increase in sleep onset latency, and dyssomnia, recorded by an increase in the mean score of the PSQI in adolescents with IA [41]. Similar associations using the PSQI have also recently been described for Indian adolescents [62], Turkish and Bangladeshi students [63,64]. A 3.25-fold decrease in the subjective assessment of sleep quality was found in Chinese adolescents with IA, compared with adolescents without addiction [65].

The mechanisms underlying the link between IA and sleep disorders have not been conclusively established [66]. The most probable is a multifactorial and two-sided model of mutual influence. Sleep disorders, reflecting psychosocial problems, depression, and anxiety-phobic disorders, can precede and contribute to the formation of IA [36]. On the other hand, sleep disorders such as insomnia can lead to the increased use of the Internet in the evening and at night, further exacerbating the problem, creating a “vicious circle”.

It is assumed that intense emotional experiences in the evening, associated with Internet activities, prevent an adolescent from falling asleep peacefully, increasing the sleep onset latency and leading to more superficial and restless sleep [56,66,67]. In this context, the hypothesis of the associative link between IA and dyssomnia, recently put forward by You et al. [68], is of interest. The authors suggest that mental rumination preceding falling asleep, as a psychological phenomenon of the automatic repeated mental experience of negative situations, analysis of their causes, feeling of inability to achieve desired goals, is characteristic of Internet-addicted adolescents and is simultaneously associated with difficulties in falling asleep and dyssomnia. There are also hypotheses of a direct physiologically determined effect of gadgets on brain activity that is unfavorable for the formation of a healthy sleep pattern. For example, it is assumed that the already mentioned effect of light from monitors on the production of melatonin, which is necessary for the formation of a healthy circadian rhythm, may underlie difficulties in falling asleep [34,35].

Disruption of a healthy night sleep pattern in Internet-addicted adolescents was accompanied in the research sample by a pronounced higher degree of daytime sleepiness, worsening due to an increase in the degree of IA, calculated by using the PDSS questionnaire. The possible explanations of this relation may be: (1) the higher rate of night activity among Internet overusers, (2) night sleep disturbances, such as insomnia, and (3) the presence of common pathogenic factors in IA and excessive daytime sleepiness, such as personality characteristics, depression, anxiety. Severe daytime sleepiness in adolescents with IA has been previously reported in a number of studies. Thus, Ekinci et al. showed a high frequency of complaints of daytime sleepiness and fatigue in Internet-addicted adolescents [38]. Demir et al. convincingly showed a positive association of the severity of daytime sleepiness with IA in university students [37].

The authors of this article managed to find only two studies in this direction, in which they also used a psychometric tool created specifically for children and adolescents—the PDSS scale. South Korean researchers have shown a pronounced association of daytime sleepiness, verified using the PDSS questionnaire, with smartphone addiction in 14–15-year-old adolescents [39]. Moreover, the risk of developing pronounced daytime sleepiness increased in parallel with an adolescent’s sleep onset latency [39]. However, only week association between sleep onset latency and PDSS score was found in our study (r = 0.194, *p* < 0.001; Table 9). In Brazilian adolescents, the PDSS scores were positively associated with the intensity of social media use and negatively—with the level of physical activity [40]. The association between PDSS score and IA score was also found in our sample, and the strength of the relationship was greater in the presence of social network addiction than in the presence of gambling addiction (r = 0.357, *p* <0.001 and r = 0.198, *p* < 0.001, respectively; Table 9).

It has been previously shown that excessive daytime sleepiness in adolescents can reduce attention and school performance, affect mood and decision-making ability, reflect the presence of anxiety-depressive disorders, and is one of the predictors of suicidal behavior [69,70,71,72]. In the authors’ opinion, the pronounced association revealed between daytime sleepiness and the severity of Internet-dependent behavior in Siberian adolescents is undoubtedly one of the negative consequences of a decrease in the quality of nighttime sleep: going to bed late, difficulty in falling asleep, frequent awakenings. On the other hand, daytime sleepiness may be one of the common manifestations of depression and anxiety-phobic disorders comorbid for IA [73,74].

The analysis of literature data shows that in the overwhelming majority of studies on sleep assessment in adolescents with IA, only one psychometric tool was used to assess addiction, and general scales were most often used, which did not identify the predominant media an adolescent consumed. Thus, in a meta-analysis by Alimoradi et al., out of the 23 included studies, only Young’s Internet Addiction Scale questionnaire was used to assess IA in 15, and in 4 only the CIAS questionnaire was used, which was also used by the authors [26]. These questionnaires reveal only the general IA pattern without reference to the consumed content. In the authors’ opinion, the undoubted advantage of this research project is the use of three tools simultaneously, which make it possible to assess not only the general, undifferentiated IA but also to identify the predominant media of addiction [75].

The presented data show that the parameters, with the highest effect size IA, regardless of the content consumed, were PDSS score and the scale of night awakenings. Daytime sleepiness was higher in all age–gender groups when using data from all three tools used to assess the IA, regardless of the presence or absence of disturbance in the night sleep pattern. These data once again confirm the above hypothesis about excessive daytime sleepiness, as a reflection of not only disturbances in the nighttime sleep schedule but also psychosocial problems comorbid for IA. We assume that emotional problems can also reflect an increase in the average score of the nighttime awakening scale, recorded in all groups, except girls with addiction to video games. On the other hand, light and restless sleep can be caused by the emotionally charged use of the Internet just before bedtime, as evidenced by an increase in sleep onset latency, especially in boys with game addiction and girls with addiction to social networks.

Regarding the nighttime sleep schedule, despite the fact of going to bed late, which was observed in both types of addiction in almost all age–gender groups, the reduction in the total time of nighttime sleep was more typical for older adolescents. Apparently, this is due to the opportunity in their schedules for younger schoolchildren to get up later, while at an older age, with increased responsibility and changes in the school schedule/the emergence of new household duties, there is no such opportunity, and when an adolescent goes to bed late, the duration of nighttime sleep is reduced. This assumption is based on family relationships traditional for Siberian adolescents and may be less obvious for other ethnic populations.

According to research data, the greatest effect on the quality of sleep was recorded in 12–14-year-olds boys with game addiction—in this group, five of the six sleep parameters used in the study showed differences between the compared groups. The least effect on sleep was recorded in the same group in case of addiction to social media—only frequent nighttime awakenings and daytime sleepiness were recorded; the nightly sleep schedule was not disturbed.

This study has some limitations. The study was not entirely anonymous; completion of questionnaires took place in a common classroom and not during an individual conversation between an interviewer and a teenager in an isolated room. The study design was based on voluntary consent. It can be assumed that some of the adolescents with psychological problems could not answer the questions truthfully and/or explicitly or implicitly evaded the survey. The above may be the reason for the social desirability bias and consent bias, which are characteristic of many psychological studies with voluntary and independent completion of questionnaires [75,76,77,78]. Therefore, future research should use more objective methods, such as an experimental design with an objective assessment of time spent on the Internet and nighttime sleep. The results of this study are presented without taking into account the ethnicity of adolescents (Russians, Khakass, Tuvans), which did not allow for assessing the effect of the ethnocultural factor on both the IA indicators and a sleep pattern, which can have significant population differences [52,79]. Such analysis is planned for the near future.

## 5. Conclusions

The following has been revealed in adolescents with IA: a habit of going to bed late and waking up late, a lower duration of nighttime sleep, a higher sleep onset latency, and frequent night awakenings, as well as more pronounced daytime sleepiness. Among the sleep parameters studied, the daytime sleepiness and nighttime awakening scales have the highest impact in Internet-addicted adolescents, regardless of the media consumed. The greatest effect on sleep quality was recorded in 12–14-year-old males with addiction to Internet games. The data obtained is advisable to use when planning psychological and pedagogical activities to normalize the sleep schedule in adolescents according to the available recommendations.

## Figures and Tables

**Table 1:** Descriptive statistics for major study variables.

Variables	All Participants	Boys	Girls	*p* (Boys vs. Girls)
Age 12–14	2228	1009 (45.3%)	1219 (54.7%)	––
Age 15–18	2387	1125 (47.1%)	1262 (52.9%)	––
Total	4615	2134 (46.2%)	2481 (53.8%)	––
CIAS results (*n* = 4615)				
Adaptive Internet use (AIU)	2390 (51.8%)	1234 (57.8%)	1156 (46.6%)	<0.001
Maladaptive Internet use (MIU)	1896 (41.1%)	788 (36.9%)	1108 (44.7%)	<0.001
Internet addiction (IA)	329 (7.1%)	112 (5.3%)	217 (8.8%)	<0.001 χ2 = 64.3, df = 2
GASA and SMDS results (*n* = 4549)				
Internet game addiction (IGA)	472 (10.4%)	328 (15.6%)	144 (5.9%)	<0.001 χ2 = 115.6, df = 1
Social media addiction (SMA)	352 (7.7%)	70 (3.3%)	282 (11.5%)	<0.001 χ2 = 102.6, df = 1
Sleep behavior characteristics				
Bedtime weekdays (in hours)	23.12 ± 1.25	23.09 ± 1.29	23.14 ± 1.22	0.258 t = −1.13
Waking up weekdays (in hours)	7.05 ± 1.26	7.14 ± 1.23	7.00 ± 1.27	<0.001 t = 4.69
Total sleep weekdays (in hours)	7.74 ± 1.57	7.90 ± 1.57	6.97 ± 1.56	<0.001 t = 6.34
Sleep onset latency (in minutes)	17.3 ± 15.9	16.1 ± 15.4	18.3 ± 16.3	<0.001 t = −4.50
Frequency of night awakenings (in points)	0.90 ± 1.00	0.75 ± 0.93	1.04 ± 1.03	<0.001 t = −9.84
PDSS score (in points)	12.0 ± 5.7	10.7 ± 5.5	13.2 ± 5.6	<0.001 t = −15.17

Note: Data are presented as *n* (%) and Mean ± SD. Pearson’s chi-squared and Student’s t tests were used, respectively.

**Table 2:** Psychometric characteristics of the questionnaires included in the study.

Questionnaire	Cronbach’s Alpha	χ^2^ df, *p*	CFI	TLI	RMSEA (90% CI)
Chen Internet Addiction Scale (CIAS)	0.909	6002 299, <0.001	0.838	0.824	0.0643 (0.0629–0.0657)
Game Addiction Scale for Adolescents (GASA)	0.876	583 14, <0.001	0.954	0.931	0.102 (0.0953–0.110)
The Social Media Disorder Scale (SMDS)	0.673	236 20, <0.001	0.931	0.903	0.0494 (0.0439–0.0552)
Pediatric Daytime Sleepiness Scale (PDSS)	0.662	485 20, <0.001	0.932	0.904	0.0716 (0.0661–0.0772)

Note: CFI—comparative fit index; TLI—Tucker–Lewis index; RMSEA—root mean square error of approximation; CI—confidence interval.

**Table 3:** Sleep parameters in adolescents aged 12–14 with IA verified with Chen Internet Addiction Scale (CIAS).

Sleep Parameters	Age 12–14											
	Boys (*n* = 1009)						Girls (*n* = 1219)					
	AIU *n* = 575	MIU *n* = 376	IA *n* = 58	*p*	d	*H*	AIU *n* = 593	MIU *n* = 534	IA *n* = 92	*p*	d	*H*
Bedtime weekdays (in hours)	23 (22–23)	23 (22–24)	23.5 (23–25)	<0.001	0.478	56.36	23 (22–23)	23 (23–24)	23 (22–24)	<0.001	0.411	51.16
Waking up weekdays (in hours)	7 (7–8)	7 (7–9)	8 (7–9)	0.008	0.176	9.69	7 (6–8)	7 (6–9)	7 (6–9)	0.016	0.144	8.28
Total sleep weekdays (in hours)	8.5 (7.7–9.5)	8.3 (7.3–9.3)	8.3 (7.3–9.1)	0.004	0.193	11.22	8.3 (7.4–9.2)	8.0 (7.0–9.0)	7.8 (6.5–9.3)	0.003	0.179	11.65
Sleep onset latency (in minutes)	10 (7–20)	10 (10–30)	15 (8–30)	0.017	0.164	8.15	12 (10–20)	15 (10–30)	15 (10–30)	<0.001	0.222	15.94
Frequency of night awakenings (in points)	0 (0–1)	1 (0–1)	1 (0–2)	0.014	0.163	8.61	1 (0–1)	1 (0–2)	1 (0–2)	<0.001	0.368	41.45
PDSS score (in points)	8 (5–12)	11 (8–15)	14 (10–18)	<0.001	0.696	111.53	10 (7–14)	14 (10–17)	17 (14–20)	<0.001	0.752	151.74

Note: Time is expressed in 24–hour clock time. AIU—adaptive Internet use; MIU—maladaptive Internet use; IA—Internet addiction. Data are presented as medians (25–75% quartiles). The Kruskal–Wallis H test was used. d—effect size (Cohen’s d) for Kruskal–Wallis *H* test calculated according to recommendations Fritz, Morris & Richler [45] with the public domain software https://www.psychometrica.de/effect_size.html (accessed on 2 October 2021).

**Table 4:** Sleep parameters in adolescents aged 15–18 with IA verified with Chen Internet Addiction Scale (CIAS).

Sleep Parameters	Age 15–18											
	Boys (*n* = 1125)						Girls (*n* = 1262)					
	AIU *n* = 575	MIU *n* = 376	IA *n* = 58	*p*	d	*H*	AIU *n* = 593	MIU *n* = 534	IA *n* = 92	*p*	d	*H*
Bedtime weekdays (in hours)	23 (22–23)	23 (23–24)	24 (23–01)	<0.001	0.571	89.61	23 (22–24)	23 (23–24)	23 (23–01)	<0.001	0.705	40.89
Waking up weekdays (in hours)	7 (6–7)	7 (6–7)	7 (6–7)	0.381	0.017	1.93	6 (6–7)	6 (6–7)	7 (6–7)	0.052	0.111	5.93
Total sleep weekdays (in hours)	7.8 (7.0–8.4)	7.2 (6.3–8.0)	6.7 (5.8–7.4)	<0.001	0.492	65.57	7.3 (6.7–8.0)	7.0 (6.2–7.8)	6.7 (5.3–7.8)	<0.001	0.334	36.13
Sleep onset latency (in minutes)	10 (7–15)	10 (7–20)	10 (9–30)	0.012	0.164	8.93	10 (8–20)	15 (10–20)	15 (10–30)	0.005	0.173	10.77
Frequency of night awakenings (in points)	0 (0–1)	1 (0–2)	1 (0–2)	<0.001	0.311	28.36	1 (0–1)	1 (0–2)	1 (0–2)	0.002	0.218	16.71
PDSS score (in points)	9 (6–13)	13 (9–16)	18 (11–22)	<0.001	0.808	159.08	11 (8–15)	15 (11–18)	18 (14–21)	<0.001	0.800	174.70

Note: Time is expressed in 24–hour clock time. AIU—adaptive Internet use; MIU—maladaptive Internet use; IA—Internet addiction. Data are presented as medians (25–75% quartiles). The Kruskal–Wallis H test was used. d—effect size (Cohen’s d) for Kruskal–Wallis *H* test calculated according to recommendations Fritz, Morris & Richler [45] with the public domain software https://www.psychometrica.de/effect_size.html (accessed on 2 October 2021).

**Table 5:** Sleep parameters in adolescents aged 12–14 with Internet game addiction (IGA) verified with Game Addiction Scale for Adolescents (GASA).

Sleep Parameters	Age 12–14									
	Boys (*n* = 1010)					Girls (*n* = 1217)				
	No IGA *n* = 833	IGA *n* = 177	*p*	d	*U*	No IGA *n* = 1130	IGA *n* = 87	*p*	d	*U*
Bedtime weekdays (in hours)	23 (22–23)	23 (23–24)	<0.001	0.332	55,363	23 (22–24)	23 (23–24)	0.020	0.133	41,849
Waking up weekdays (in hours)	7 (7–8)	8 (7–9)	<0.001	0.262	58,691	7 (6–8)	7 (6–9)	0.120	0.089	44,359
Total sleep weekdays (in hours)	8.4 (7.6–9.4)	8.3 (7.3–9.3)	0.190	0.083	68,104	8.2 (7.2–9.1)	7.9 (6.5–9.3)	0.461	0.042	46,784
Sleep onset latency (in minutes)	10 (7–20)	15 (10–30)	0.013	0.164	54,968	15 (10–25)	20 (13.5–30)	<0.001	0.222	31,964
Frequency of night awakenings (in points)	0 (0–1)	1 (0–2)	0.015	0.175	63,659	1 (0–2)	1 (0–2)	0.350	0.051	43,895
PDSS score (in points)	9 (6–13)	13 (10–17)	<0.001	0.561	44,680	12 (8–16)	16 (13–19)	<0.001	0.340	30,834

Note: Time is expressed in 24–hour clock time. AIU—adaptive Internet use; MIU—maladaptive Internet use; IA—Internet addiction. Data are presented as medians (25–75% quartiles). The Mann–Whitney *U* test was used. d—effect size (Cohen’s d) for Mann–Whitney *U* test calculated according to recommendations Fritz, Morris & Richler [45] with the public domain software https://www.psychometrica.de/effect_size.html (accessed on 2 October 2021).

**Table 6:** Sleep parameters in adolescents aged 15–18 with Internet game addiction (IGA) verified with Game Addiction Scale for Adolescents (GASA).

Sleep Parameters	Age 15–18									
	Boys (*n* = 1045)					Girls (*n* = 1258)				
	No IGA *n* = 985	IGA *n* = 156	*p*	d	*U*	No IGA *n* = 1203	IGA *n* = 59	*p*	d	*U*
Bedtime weekdays (in hours)	23 (22–24)	24 (23–24)	<0.001	0.260	60,184	23 (22–24)	24 (23–01)	0.002	0.177	26,952
Waking up weekdays (in hours)	7 (6–7)	7 (6–7)	0.393	0.051	71,573	6 (6–7)	7 (6–7)	0.006	0.154	28,598
Total sleep weekdays (in hours)	7.6 (6.8–8.2)	7.0 (6.0–7.9)	<0.001	0.250	58,900	7.2 (6.4–7.9)	6.7 (5.3–8.0)	0.068	0.103	30,400
Sleep onset latency (in minutes)	10 (7–20)	10 (7–20)	0.313	0.062	62,948	10 (10–20)	15 (7–30)	0.467	0.042	28,901
Frequency of night awakenings (in points)	0 (0–1)	1 (0–2)	<0.001	0.222	62,719	1 (0–2)	1 (0–2)	0.177	0.076	32,146
PDSS score (in points)	10 (7–14)	14 (10–18)	<0.001	0.417	51,691	13 (10–17)	17 (13–22)	<0.001	0.302	21,590

Note: Time is expressed in 24–hour clock time. AIU—adaptive Internet use; MIU—maladaptive Internet use; IA—Internet addiction. Data are presented as medians (25–75% quartiles). The Mann–Whitney *U* test was used. d—effect size (Cohen’s d) for Mann–Whitney *U* test calculated according to recommendations Fritz, Morris & Richler [45] with the public domain software https://www.psychometrica.de/effect_size.html (accessed on 2 October 2021).

**Table 7:** Sleep parameters in adolescents aged 12–14 with social media addiction (SMA) verified with the Social Media Disorder Scale (SMDS).

Sleep Parameters	Age 12–14									
	Boys (*n* = 1010)					Girls (*n* = 1217)				
	No SMA *n* = 966	SMA *n* = 44	*p*	d	*U*	No SMA *n* = 1050	SMA *n* = 167	*p*	d	*U*
Bedtime weekdays (in hours)	23 (22–24)	23 (22–24)	0.425	0.050	19,742	23 (22–24)	23 (22–24)	0.010	0.142	77,249
Waking up weekdays (in hours)	7 (7–8)	7 (7–9)	0.639	0.030	19,797	7 (6–8)	7 (6–9)	0.293	0.058	84,471
Total sleep weekdays (in hours)	8.4 (7.5–9.4)	8.2 (7.5–9.3)	0.874	0.010	19,786	8.2 (7.2–9.1)	8.0 (7.0–9.1)	0.612	0.029	85,456
Sleep onset latency (in minutes)	10 (7–20)	15 (10–30)	0.095	0.110	14,941	15 (10–25)	15 (10–30)	0.015	0.058	67,908
Frequency of night awakenings (in points)	0 (0–1)	1 (0–2)	0.002	0.194	15,762	1 (0–2)	1 (0–2)	<0.001	0.243	68,059
PDSS score (in points)	9 (6–13)	12 (10–17.5)	<0.001	0.251	15,569	12 (8–16)	16 (12–20)	<0.001	0.480	53,969

Note: Time is expressed in 24–hour clock time. AIU—adaptive Internet use; MIU—maladaptive Internet use; IA—Internet addiction. Data are presented as medians (25–75% quartiles). The Mann–Whitney *U* test was used. d—effect size (Cohen’s d) for Mann–Whitney *U* test calculated according to recommendations Fritz, Morris & Richler [45] with the public domain software https://www.psychometrica.de/effect_size.html (accessed on 2 October 2021).

**Table 8:** Sleep parameters in adolescents aged 15–18 with social media addiction (SMA) verified with the Social Media Disorder Scale (SMDS).

Sleep Parameters	Age 15–18 лeт									
	Boys (*n* = 1141)					Girls (*n* = 1262)				
	No SMA *n* = 1113	SMA *n* = 28	*p*	d	*U*	No SMA *n* = 1144	SMA *n* = 118	*p*	d	*U*
Bedtime weekdays (in hours)	23 (22–24)	24 (23–01)	0.002	0.175	10,502	23 (22–24)	23 (23–24)	0.081	0.094	61,213
Waking up weekdays (in hours)	7 (6–7)	7 (6–7)	0.824	0.013	14,597	6 (6–7)	6 (6–7)	0.662	0.022	66,645
Total sleep weekdays (in hours)	7.5 (6.7–8.2)	6.9 (5.5–7.8)	0.008	0.158	10,485	7.2 (6.3–7.9)	7.0 (5.9–7.8)	0.096	0.094	60,999
Sleep onset latency (in minutes)	10 (7–20)	10 (9–20)	0.482	0.044	11,223	10 (10–20)	15 (10–27.5)	0.053	0.111	51,979
Frequency of night awakenings (in points)	0 (0–1)	1.5 (0–2)	0.004	0.140	11,472	1 (0–2)	1 (1–2)	<0.001	0.223	52,376
PDSS score (in points)	10 (7–15)	16 (12.5–22.5)	<0.001	0.297	7061	13 (10–17)	17 (13–21)	<0.001	0.356	45,302

Note: Time is expressed in 24–hour clock time. AIU—adaptive Internet use; MIU—maladaptive Internet use; IA—Internet addiction. Data are presented as medians (25–75% quartiles). The Mann–Whitney *U* test was used. d—effect size (Cohen’s d) for Mann–Whitney *U* test calculated according to recommendations Fritz, Morris & Richler [45] with the public domain software https://www.psychometrica.de/effect_size.html (accessed on 2 October 2021).

**Table 9:** Correlation matrix of studied variables.

Variables	Sleep Onset Latency	Total Night Sleep	PDSS Score	CIAS Score	GASA Score	SMDS Score
Sleep onset latency	–					
Total night sleep	–0.106	–				
PDSS score	0.194	–0.311	–			
CIAS score	0.135	–0.173	0.422	–		
GASA score	0.074	*–0.027*	0.198	0.415	–	
SMDS score	0.121	–0.111	0.357	0.608	0.266	–

Note: all values are significant at *p* < 0.001, except for underlined values (*p* > 0.05).

## Data Availability

The datasets generated for this study are available on request to the corresponding author.

## Abbreviations

IA	Internet addiction
CIAS	Chen Internet Addiction Scale
GASA	Game Addiction Scale for Adolescents
SMDS	The Social Media Disorder Scale
PSQI	Pittsburgh Sleep Quality Index
PDSS	Pediatric Daytime Sleepiness Scale
IGA	Internet game addiction
MIU	maladaptive Internet use
ADHD	attention deficit–hyperactivity disorder
SMA	social media addiction
SD	standard deviation
CFI	comparative fit index
TLI	Tucker–Lewis index
RMSEA	root mean square error of approximation
CI	confidence interval
